# Development and Validation of the Bullied Cognitions Inventory (BCI)

**DOI:** 10.1007/s10608-023-10412-6

**Published:** 2023-09-07

**Authors:** Belinda Graham, Anke Ehlers

**Affiliations:** Department of Experimental Psychology, Oxford Centre for Anxiety Disorders and Trauma, University of Oxford, The Old Rectory, Paradise Square, Oxford, OX1 1TW UK

**Keywords:** Cognitions, Bullying, PTSD, Social anxiety

## Abstract

**Background:**

Bullying increases risk of social anxiety and can produce symptoms of posttraumatic stress disorder (PTSD). According to cognitive models, these are maintained by unhelpful beliefs, which are therefore assessed and targeted in cognitive therapy. This paper describes psychometric validation of a new measure of beliefs related to bullying experiences.

**Methods:**

In an online survey of 1879 young people before starting university or college in the UK, 1279 reported a history of bullying (N = 1279), and 854 rated their agreement with beliefs about self and others related to bullying experiences and completed symptom measures of social anxiety and PTSD related to bullying. An empirical structure for a Bullied Cognitions Inventory was established using exploratory and confirmatory factor analyses and assessed using model fit statistics and tests of reliability and validity.

**Results:**

Fifteen items clustered into four themes: “degraded in the eyes of others”, “negative interpretations of reactions to bullying”, “recognisable as a bullying victim” and “social defeat”. The measure has acceptable reliability and validity and, accounting for existing cognitive measures, explained additional variance in symptoms of PTSD but not social anxiety.

**Conclusions:**

The Bullied Cognitions Inventory (BCI) is a valid and reliable tool for measuring cognitions related to bullying. It may be useful in therapy for identifying and monitoring unhelpful cognitions in those who were bullied.

**Supplementary Information:**

The online version contains supplementary material available at 10.1007/s10608-023-10412-6.

## Introduction

Bullying is associated with higher likelihood of social anxiety disorder (SAD; Siegel et al., 2009; Storch et al., 2005) and can produce symptoms of posttraumatic stress disorder (PTSD; Idsoe et al., 2021; Nielsen et al., 2015). SAD is characterised by excessive fear or anxiety about social situations in which the individual may feel judged by other people (American Psychiatric Association, 2013; World Health Organisation, 2019) and which are subsequently avoided or endured with intense fear or anxiety which is disproportionate to the actual threat posed. Following a traumatic experience, PTSD symptoms may develop that include distressing unwanted memories of the trauma, avoidance of reminders, negative alterations in cognitions and mood, and alterations in arousal and reactivity (American Psychiatric Association, 2013; World Health Organisation, 2019). Cognitive models suggest that negative beliefs maintain social anxiety (Clark & Wells, 1995) and PTSD (Ehlers & Clark, 2000) and are commonly connected in meaning to past negative social experiences (SAD) or trauma (PTSD). This paper presents the development and psychometric validation of a Bullied Cognitions Inventory (BCI) that measures beliefs about self and others related to bullying experiences and reports associations with SAD and PTSD among young people who have been bullied.

Bullying is a pervasive form of interpersonal aggression (Craig et al., 2009; Skrzypiec et al., 2018) including behaviours that are overt (e.g., name calling, making sexual comments or gestures at someone, or hitting, shoving, physically aggressing) or relational (e.g., excluding somebody from a social group, ignoring them, gossiping behind their backs) that can happen in person and online, with considerable overlap (Cosma et al., 2020). Bullying behaviour is distinguished from conflict or other aggressive acts by characteristics of intentionality, frequency, and power differential (i.e., the behaviour is deliberately hurtful, is not a one-off attack, and is perpetrated by those with higher status or power over those with less). Power imbalance is thought to set bullying apart from disagreements, teasing between friends, playful fighting, or general hostility between individuals or groups and may be of particular interest when evaluating appraisals of bullying experiences and their impact on mental health. There is evidence indicating that power imbalance exacerbates adjustment problems associated with victimisation (Malecki et al., 2014). Furthermore, the presence of a power imbalance between the perpetrator and the target echoes interpersonal trauma and abuse (such as physical or sexual assault) that can precede stressor related disorders including PTSD. Evaluating the relationship between perceived power differential during bullying experiences and later psychopathology is potentially clinically important and may explain some of the variance in PTSD symptoms among people who have been bullied. Therefore, in this study power imbalance was conceptualised as an additional cognitive variable rather than an inclusion criterion for bullying, so that its contribution as a potential cognitive predictor of psychological adjustment could be investigated.

There is increasing evidence that negative psychosocial effects of bullying in childhood and adolescence can persist into adulthood (Arseneault, 2018; McDougall & Vaillancourt, 2015) and recognition that these experiences need to be meaningfully incorporated into mental health assessment and treatment. Peer victimisation in childhood and adolescence is associated with later social anxiety (Siegel et al., 2009; Storch et al., 2005). Adults with social anxiety are particularly likely to report having been bullied or teased when they were younger (McCabe et al., 2003, 2010) and socially anxious students recall higher levels of social exclusion and relational victimisation (Boulton, 2013). Bullying is also associated with PTSD symptoms among adolescents (Crosby et al., 2010; Guzzo et al., 2014; Mynard et al., 2000; Storch & Esposito, 2003) and students in higher education (Andreou et al., 2021; Manrique et al., 2020), and the association persists into adulthood (Idsoe et al., 2021; Nielsen et al., 2015). Of note, over a quarter of sexual minority adults who self-identified as victims of homophobic bullying at school continued to be distressed regularly by recollections of their experiences (Rivers, 2006). Given the developmental context and repetitive nature of experiences, bullying is a particularly potent context for social trauma, which is described in more detail below.

There is longstanding debate regarding the definition and measurement of psychological trauma (Weathers & Keane, 2007) in relation to PTSD diagnosis (Brewin et al., 2009). Experiencing threatened death, serious injury, or sexual violence (DSM-5) or exposure to an extremely threatening or horrific event or series of events (ICD-11) is a prerequisite for developing stress related disorders including PTSD (American Psychiatric Association, 2013; World Health Organisation, 2019). This means that PTSD cannot be diagnosed in the absence of this type of event, although symptoms consistent with PTSD may be present. Socially traumatic events have been defined as extremely unpleasant social events during which the individual experiences intense anxiety and perceives ridicule or rejection by others (Wild & Clark, 2011) and can be perceived as extremely threatening to the social self (Idsoe et al., 2021). Socially traumatic experiences occur in many environments (e.g., work meetings, performance events) but occur particularly frequently and repetitively in the context of bullying or victimisation by peers (Wild & Clark, 2011). Socially traumatic experiences are associated with social anxiety in adolescents (Pontillo et al., 2019) and adults (McCabe et al., 2010) and can produce symptoms of posttraumatic stress disorder (Bjornsson et al., 2020; Carleton et al., 2011) but they are not qualifying events for PTSD diagnosis.

There is evidence for psychological common ground between reactions to threats to physical and social self among clinical populations. In a sample of 75 individuals with and without SAD (Erwin et al., 2006), socially stressful events were common (100% of those with SAD, and 70% of those without) but more than a third of those with SAD, compared with none in the control group, experienced PTSD-like symptoms in connection to the stressful event, including distressing and persistent intrusive thoughts and feelings. Similarly, among 60 individuals seeking treatment for SAD, a third of those who had experienced social trauma reported PTSD-like symptoms (Bjornsson et al., 2020). The authors suggested that individuals with SAD may be more likely to interpret stressful social events as traumatic. Individuals with SAD have also reported higher skin conductance response to social trauma memories (Sansen et al., 2015). Indeed, commonalities exist in the phenomenology of PTSD and SAD, in terms of avoidance of related stimuli (social interactions, relationships) and negative cognitions related to past experiences, the self and other people. In response to socially traumatic experiences, individuals may experience fear of negative evaluation (consistent with SAD), as well as distressing intrusive memories of the experience, physiological symptoms when reminded of it, feelings of detachment, restricted range of affect, and hyperarousal expressed as irritability, outbursts or difficulty concentrating (consistent with PTSD), and avoidance of social situations (consistent with both SAD and PTSD). It is possible that individuals experiencing social anxiety as well as PTSD-like symptoms in response to social trauma may be best understood not as suffering with two distinct disorders but rather as experiencing a stress response to the social experience that involves an ongoing sense of social threat associated with intrusive memories, hypervigilance and avoidance related to their experience.

Cognitive models propose that maladaptive beliefs maintain social anxiety disorder (Clark & Wells, 1995) and PTSD (Ehlers & Clark, 2000). Results from cross-sectional and prospective studies support this hypothesis (e.g., SAD: Clark, 2005; PTSD: Beierl et al., 2019). Cognitive therapy for SAD (Clark et al., 2003, 2006) and PTSD (Ehlers et al., 2005, 2014) target idiosyncratic beliefs relevant to the disorder and have been shown to be highly effective. Clark and Wells (1995) propose that social anxiety symptoms persist due to overestimating social threat based on beliefs about unfavourable personal characteristics and negative expectations of others’ reactions. Unhelpful attempts to manage the threat maintain the problem. Common negative appraisals are often connected in meaning to past experiences of humiliation or rejection (Wild et al., 2007) and relate to performance in current social situations (e.g., “I always say the wrong thing”, “I am boring”). Similarly, Ehlers and Clark (2000) suggest that an ongoing sense of external or internal current threat is central to PTSD. One source of the perceived threat is excessively negative appraisals about trauma that lead to negative emotions (such as fear, anger, guilt, shame, disgust) and motivate unhelpful coping strategies. These inadvertently maintain the problem. Appraisals commonly relate to the traumatic experience (e.g., “bad things always happen to me”, “others will look down on me as they can see that I am a victim”) and its sequelae (e.g., “I’ll never be able to trust people again”, “I’m going mad”). Changes in these beliefs have been shown to mediate symptom change in both conditions (SAD: Thew et al., 2020; PTSD: Kleim et al., 2013) and frequent assessment of cognitions to guide treatment and monitor progress in cognitive therapy is recommended (Warnock-Parkes et al., 2020; Wild et al., 2020). Ehlers et al. (1998) observed that during prolonged and repeated interpersonal trauma, some people experience a state of mental defeat where they perceive a complete loss of autonomy, degradration and dehumanisation. Mental defeat has been shown to predict PTSD after interpersonal trauma (Ehlers & Clark, 2000; Kleim et al., 2007). It is conceivable that perceived defeat also plays a role in bullying.

Cognitions related to memories of socially traumatic events can contribute to onset and maintenance of social anxiety (Hackmann et al., 2000; Norton & Abbott, 2017; Pontillo et al., 2019; Wild & Clark, 2011) and are important therapeutic targets. Bullies may confirm negative beliefs repeatedly and explicitly (e.g., telling the person directly that they are boring/weird/unacceptable) making these experiences particularly potent. Appraisals of self and others and personal reactions related to experiences of bullying are associated with psychological adjustment (Troop-Gordon & Ladd, 2005) and may contribute to distress, avoidance and shame (Wu et al., 2021). Victimisation may be associated with beliefs of being less than peers (Carlisle & Rofes, 2007) and increased self-criticism (Kopala-Sibley et al., 2013). Social anxiety and poorer psychosocial adjustment into adulthood may also be fuelled by shame (Strøm et al., 2018) and beliefs of deserving or being responsible for childhood victimisation (Boulton, 2013). Indeed, after bullying, new social experiences may be misinterpreted as excessively threatening (Fox et al., 2022) including via heightened attention to negative social cues and interpreting social situations and others’ intentions as more negative, hostile, or dangerous (Kellij et al., 2022).

For PTSD, the Posttraumatic Cognitions Inventory (PTCI; Foa et al., 1999) includes items that measure negative thoughts about the self, the world, and self-blame. For social anxiety, the Social Attitudes Questionnaire (SAQ; D.M. Clark, 2005) measures problematic beliefs about social behaviour, social situations and patient’s evaluations of their social self. These measures are important tools in cognitive therapy but were not developed within bullied samples. Specific bullying-related cognitions may be particularly important for maintaining the sense of ongoing threat that perpetuates SAD and PTSD symptoms related to bullying experiences. A bespoke measure tailored for people who were bullied could provide specific relevant prompts for therapists, aid disclosure, and provide a replicable basis for research into specific cognitions maintaining emotional difficulties. Therefore, potential items for this measure were developed and piloted following literature review, consultations with expert clinicians, and qualitative analyses of interviews with young people who were bullied (see Graham, 2022).

This study describes psychometric evaluation of a new measure of cognitions relevant to SAD and PTSD among a large sample of young people who were bullied. An empirical structure among potential items was established using exploratory and confirmatory factor analyses (Brown, 2015) and assessed using model fit statistics (Sellbom & Simms, 2019) and multiple tests of reliability and validity.

## Methods

### Procedure

Young people aged 18 to 29 expecting to start university or college in September 2018 were contacted by email using a student networking app mailing list distributed by the Universities and Colleges Application System (UCAS) and available free of charge to all UK higher education applicants. Those who chose to participate completed an online survey about bullying and mental health via Qualtrics (qualtrics.com). They were encouraged to take breaks if needed but to complete the questionnaires on one day. Since the survey included emotionally difficult themes, information about support services was offered to all participants and an automatically generated email was sent 24 h after completing the survey to check-in regarding wellbeing and offer additional options for support. Participants were compensated with entry into a prize draw for online shopping vouchers. Participants were invited to complete a shorter survey 1 week later, including repeated measures for test–retest reliability. The survey was closed when ten percent of responses had been completed. The study received all relevant ethical approvals.

### Measures

#### Background Characteristics

Participants provided their age, gender, ethnicity, and sexual orientation.

#### Item Pool for Bullied Cognitions Inventory

Participants rated their agreement with 23 statements about self and others related to bullying experiences over the last month on a 7-point Likert scale from 1 *(totally disagree)* to 7 *(totally agree)* where higher scores indicate more maladaptive appraisals. Items were based on qualitative analysis of interviews with young people who were bullied, literature review, and consultation with expert clinicians.

#### Posttraumatic Cognitions Inventory

The PTCI (Foa et al., 1999) measures excessively negative appraisals of a traumatic experience and its consequences. In this study an adapted 20-item version (Kleim et al., 2013) referred to the worst bullying experience as the index event. Negative cognitions about the self, the world and self-blame are rated on a 7-point Likert scale from 1 *(totally disagree)* to 7 *(totally agree)*. A mean score is calculated (range 1–7) and higher scores indicate more maladaptive appraisals. Internal consistency in this sample was excellent, $\alpha$ = .94. It can be downloaded from https://oxcadatresources.com.

#### Social Attitudes Questionnaire (SAQ)

The SAQ (Clark, 2005) is a measure of beliefs about the self and social interactions that are thought to make an individual vulnerable to social anxiety disorder. It is a 50-item scale that includes beliefs about excessively high standards for social performance, conditional beliefs, and unconditional beliefs that are rated on a 7-point Likert scale from 1 *(totally agree)* to 7 *(totally disagree)*. A mean score is calculated (range 1–7) and higher scores indicate lower endorsement of unhelpful beliefs. Internal consistency in this sample was excellent, $\alpha$ = .98. It can be downloaded from https://oxcadatresources.com. This was completed by the retest subsample only (*n* = 133).

#### Victimisation

The California Bullying Victimization Scale-Revised (Reid et al., 2016) asks if the participant has experienced any of nine types of direct and indirect bullying experiences, including over the internet in a mean or hurtful way during their life. Participants selected the “worst” type of bullying they experienced and rated the frequency each type occurred, “*a few times a year or less*”, “*about once a month*”, “*2 or 3 times a month*”, “*about once a week*”, “*several times a week*”. Power imbalance was inferred when the aggressor was reported to be older, bigger, more athletic, attractive, popular, wealthy, or intelligent. The threshold for bullying was defined as reporting five or more types of bullying (reflecting range of experiences) and/or frequency 2 to 3 times per month or more (reflecting repeated exposure).

#### Psychopathology

PTSD symptom severity was measured using the PTSD Checklist for DSM-5 (PCL-5; Weathers et al., 2013). Participants answered in relation to their worst bullying experience and rated on a 5-point Likert scale of 0 (*not at all*) to 4 (*extremely*) how much they have been bothered by each symptom over the last month (range 0–80). Social anxiety symptom severity was measured using the Social Phobia Inventory (SPIN; Connor et al., 2000). Participants rated on a 5-point Likert scale of 0 (*not at all*) to 4 (*extremely*) how much each statement applied over the past week (range 0–68). Depression symptom severity was measured using the Patient Health Questionnaire-9 (PHQ-9; Kroenke et al., 2001). Participants rated on a 4-point Likert scale of 0 (*not at all*) to 4 (*nearly every day*) how often they had been bothered by each problem over the last 2 weeks, with higher scores indicating more severe depression (range 0–27). General anxiety symptom severity was measured using the GAD-7 (Spitzer et al., 2006). Participants rated on a 4-point Likert scale of 0 (*not at all*) to 4 (*nearly every day*) how often they were bothered by each problem over the last 2 weeks (range 0–21). On each scale, higher scores indicate more severe symptoms.

### Data Analysis

Due to online collection method, data were checked for challenges to reliability indicated by “straight-lining” (repeatedly selecting the same response across the survey) and low total response time compared with number of questionnaires completed (time automatically recorded by Qualtrics). No participants were excluded because of these checks. Cases were included if they responded to at least one item in the pool. Data was analysed using SPSS 27 and R (R Core Team, 2022), using RStudio (RStudio Team, 2022) with haven (Wickham et al., 2022b), tidyr (Wickham & Girlich, 2022), dplyr (Wickham et al., 2022a), psych (Revelle, 2021) and lavaan (Rosseel, 2012; Rosseel et al., 2022).

#### Item Reduction and Factor Analyses

Sampling adequacy for the item pool was confirmed by Kaiser–Meyer–Olkin (KMO) score greater than 0.7 (Kaiser & Rice, 1974) and a significant result on Bartlett’s test of sphericity. The sample was split into two random half datasets using R’s random sampling function for model development (EFA, n = 650) and validation (CFA, n = 629). Exploratory factor analysis (EFA) determines the smallest number of interpretable factors needed to explain the correlations among a set of items. Items were considered for removal if they had weak factor loadings (< 0.4) or high cross-loading across factors indicated by high complexity rating, low difference in factor loading (< 0.2) or two significant loadings (> 0.3). EFA was repeated after each item was removed. Confirmatory factor analysis (CFA) tests the suggested factor structure in a different sample.

Factor extraction was guided by multiple metrics (Finch, 2019) namely number of factors with Eigenvalues > 1 (Guttman–Kaiser rule), number of factors indicated before the elbow on a scree plot, number of factors suggested by parallel analysis, and comparisons of model fit. There was little missing data (completion rate 0.995–0.998) but skewness was high (common when assessing potentially clinical features in a non-clinical sample) and inter-factor correlation was expected. Therefore, an oblique (oblimin) rotation was used and MLR (maximum likelihood robust) estimation was applied, which is robust to non-normality. Model fit was assessed according to four recommended fit statistics (Brown, 2015; Hu & Bentler, 1999). Chi-square fit was determined such that $\chi^{2}$:df ratio below 3:1 is acceptable (i.e., $\chi^{2}$/df below 3). On the Comparative Fit Index (CFI), Tucker Lewis Index (TLI), and root mean square error of approximation (RMSEA), acceptable fit was indicated by CFI ≥ .90, TLI ≥ .90, RMSEA ≤ .08, and good fit was indicated by CFI ≥ .95, TLI ≥ .95, and RMSEA ≤ .06.

Once a suitable structure was defined in the EFA sample, the same structure was applied within the CFA sample and model fit was evaluated using the same indices and using the highest loading item as the reference item for each factor. To assess the appropriateness of using single scale scores, fit statistics were evaluated for each full scale using a single second-order factor. Modification indices represent the improvement in model $\chi^{2}$ that is achieved by freeing the residual variance correlation between two items and were considered only if very large (> 30) and conceptually appropriate (Brown, 2015).

#### Reliability and Validity

Several tests of reliability (internal consistency, test–retest) and validity (convergent, discriminant, criterion) were applied to evaluate the robustness of the scales. Internal consistency was indicated by Cronbach’s alpha for extracted factors and the full scale, 0.7 to 0.8 (acceptable), 0.8 to 0.9 (very good), > 0.9 (potential multicollinearity). Test–retest reliability was indicated by a correlation 0.40–0.59 (fair), 0.60–0.74 (good) and > 0.75 (excellent). Convergent validity was indicated by the percentage of item variance explained by the extracted factors with a minimum threshold of 0.5, meaning that 50% of item variance is explained by all extracted factors (Hair et al., 2010). Discriminant validity between factors (representing subscales) indicated the extent to which they reflect distinct constructs such that factor correlations < 0.85 (acceptable) with higher correlations indicating potential multicollinearity.

Criterion validity was indicated by Pearson correlations with symptoms of PTSD to bullying experience (PCL-5) and Social Anxiety (SPIN) and cognitive measures (PTCI, SAQ). To assess the unique contributions of the new measures to explaining variance in the outcomes, simultaneous multiple logistic regressions included the new measures alongside pre-existing measures of cognitions, and perceived power imbalance during the event.

Finally, for diagnostic group comparisons, likely diagnosis of SAD was inferred from SPIN sum score 19 or above (Connor et al., 2000) and likely diagnosis of PTSD was inferred from PCL sum score 31 or above (Weathers et al., 2013). Participants were categorised into four groups: no likely diagnosis of either PTSD or SAD (PCL < 31, SPIN < 19), likely diagnosis of SAD only (PCL < 31, SPIN ≥ 19), likely diagnosis of PTSD only (PCL ≥ 31, SPIN < 19), and likely diagnosis of both SAD and PTSD (SPIN ≥ 19, PCL ≥ 31). Group comparisons were conducted using multinomial logistic regressions with likely diagnostic status as the dependent variable. Overall model statistics were reviewed for goodness of fit with the data and then comparisons were calculated between each of the four outcome categories (None, SAD, PTSD, SAD + PTSD) by setting each in turn as the reference group.

## Results

### Participants

Of 1879 young people who took part in the survey, 1279 reported having been bullied. Survey participants were randomly allocated into two groups of 650 and 629 for the purposes of data analyses. Of these 449 and 405 respectively provided data for at least one potential BCI item and were included in these analyses. Participant characteristics are presented in Table 1. The two groups did not differ in terms of bullying exposure, age, gender, ethnicity, sexual orientation, or PTSD. There was a small difference (d = 0.15) in social anxiety severity in that the EFA sample reported slightly greater severity. There was large variability in SPIN scores in both samples with standard deviations > 16.

**Table 1:** Participant characteristics for bullied cognitions inventory EFA/CFA samples

	EFA sample (*n* = 449)	CFA sample (*n* = 405)	Statistic (*t*, *χ*^*2*^)
Age in years M* (SD)*	19.42 (2.41)	19.39 (2.35)	*t* = 0.233
Gender n (*%*)			
Female	347 (77.3)	288 (71.1)	*χ*^*2*^ = 5.022
Male	91 (20.3)	105 (25.9)	
Other	11 (2.4)	6 (1.5)	
Prefer not to say	0 (0.0)	6 (1.5)	
Sexual orientation n* (%)*			
Heterosexual	314 (69.9)	258 (63.7)	*χ*^*2*^ = 6.856
Bisexual	76 (16.9)	76 (18.8)	
Homosexual	16 (3.6)	29 (7.2)	
Other/unsure	40 (8.9)	36 (8.9)	
Prefer not to say	3 (0.7)	6 (1.5)	
Ethnicity n *(%)*			
White	369 (82.2)	323 (79.8)	*χ*^*2*^ = 0.940
Other	77 (17.1)	80 (19.8)	
Prefer not to say	3 (0.7)	2 (0.5)	
Type of bullying experienced n (*%*)			
Teased	422 (94.0)	384 (94.8)	
Ignored	409 (91.1)	358 (88.4)	
Gossiped about	393 (87.5)	347 (85.7)	
Sexual comments	305 (67.9)	270 (66.7)	
Threatened	264 (58.8)	234 (57.8)	
Hit	251 (55.9)	236 (58.3)	
Online	225 (50.1)	211 (52.1)	
Belongings stolen	189 (42.1)	164 (40.5)	
Other	50 (11.1)	41 (10.1)	
Number of types experienced M* (SD)*	5.59 (1.87)	5.54 (1.92)	*t* = 0.328
Social anxiety M* (SD)*	32.68 (16.74)	30.28 (16.33)	*t* = 2.119
PTSD M* (SD)*	27.57 (19.35)	29.46 (19.72)	*t* = − 1.412
Depression M* (SD)*	11.53 (7.12)	11.49 (7.13)	*t* = 0.101
General anxiety M* (SD)*	9.17 (6.07)	8.95 (6.04)	*t* = 0.528

All participants had experienced five or more types of bullying and/or bullying 2/3 times per month or more. Statistic refers to comparison between EFA and CFA samples. Social anxiety (SPIN; range 0–68). PTSD to worst bullying experience (PCL-5; range 0–80). Depression (PHQ-9; range 0–27). General anxiety (GAD-7; range 0–21). Group differences do not include prefer not to say category

*EFA* exploratory factor analysis, *CFA* confirmatory factor analysis

### Item Reduction

Of the original 23 items considered for inclusion in the measure, five were removed due to extreme skewness (1,*“I deserve to be treated the way they treated me”*), very low correlation with main outcomes (7, *“If someone is more important than me then they have the right to pick on me”*), repetitive wording with another item (10,*“If people knew I was picked on they would find me less attractive”; retained 13*), unclear concept (18,*“I wish I had physical scars so people could see how bad it was”*) and unclear wording (3, *“I am the classic example of someone who is easy to pick on”*). The remaining 18 items were appropriate for factor analysis evidenced by high Kaiser–Meyer–Olkin sampling adequacy (0.96) and significant Bartlett’s test of sphericity ($\chi^{2}$ = 6,334.33, df = 171, *p* < .001). Inter-item and item-total correlations for all original items are included as Supplementary materials.

### Exploratory Factor Analysis

Items were entered into an EFA among the first random half of the sample (*n* = 449) and the suggested number of factors varied according to method used. Eigenvalues suggested a 2-factor solution (10.68, 1.24, 0.84), scree plot suggested up to three factors, parallel analysis suggested 4 factors. Therefore, model fit and factor loadings in the EFA were evaluated for two, three and four factor solutions to establish a well-fitting and conceptually coherent model. Fit statistics indicated poor fit for the two factor solution (TLI = 0.90, RMSEA = 0.09, $\chi^{2}$ = 628.24 on df = 134, $\chi^{2}$:df = 4.69), marginally acceptable fit for the three factor solution (TLI = 0.93, RMSEA = 0.07, $\chi^{2}$ = 390.84 on df = 117, $\chi^{2}$:df = 3.34), and excellent fit for the four factor solution (TLI = 0.96, RMSEA = 0.06, $\chi^{2}$ = 245.48 on df = 101, $\chi^{2}$:df = 2.43).

Items with relatively low factor loadings (< 0.4) and/or high complexity indicated by high cross-loadings (within 0.2) were considered for deletion. This applied across all factor solutions for item 2, *“If I fail at anything it means the person or people who picked on me were right”*. After removing this item, model fit for the three-factor solution remained poor, but the four-factor model fit improved, with good conceptual fit supported by a two-item factor of the items *“I have been totally degraded”* and *“I have been totally humiliated”*. Although factors can be indicated by two items in EFA, particularly if highly correlated as the items are here, this would produce an unidentified model in subsequent CFA, where factors must include three or more items to produce a stable scale. Therefore, despite relatively low loading onto this factor and high cross loading, item 5, *“If I hadn’t been picked on maybe I would be a better person than I am now”* was retained to support the overall stability of the measure. Two further items were removed to simplify the scale without reducing model fit (6, *“I still believe the things that other people told me about myself”* and 9,*“If people knew I was picked on they would reject me”*).

Among the remaining 15 items, fit statistics indicated poor fit for the two-factor solution (TLI = 0.89, RMSEA = 0.10, $\chi^{2}$ = 403.55 on df = 76, $\chi^{2}$:df = 5.31) acceptable fit for the three-factor solution on all but one of the indicators (TLI = 0.92, RMSEA = 0.08, $\chi^{2}$ = 258.11 on df = 63, $\chi^{2}$:df = 4.10) and a much closer fit to the data for the four-factor solution (TLI = 0.97, RMSEA = 0.05, $\chi^{2}$ = 111.36 on df = 51, $\chi^{2}$:df = 2.18) which also produced coherent and interpretable factors. Of note, removing item 5 which was retained to stabilise the four-factor solution did not improve model fit for the three-factor solution (TLI = 0.911, RMSEA = 0.092, $\chi^{2}$ = 250.47 on df = 52, $\chi^{2}$:df = 4.82). Therefore, the four-factor model with 15 items was chosen. This model explained 52.5% of the variance and had adequate factor loadings for all factors. The factors had good conceptual fit, described as *“degraded in the eyes of others”* (3 items, 12.0%), *“negative interpretations of reactions to bullying”* (6 items, 18.6%), *“recognisable as a bullying victim”* (3 items, 12.3%), and *“social defeat”* (3 items, 9.4%). The total score range is 15 to 105.

### Confirmatory Factor Analysis

The four-factor structure was evaluated using CFA in the second random half of the sample (*n* = 405). The four-factor model had acceptable fit for the data (CFI = 0.94, TLI = 0.92, RMSEA = 0.08 (CI 0.07–0.09), $\chi^{2}$ = 299.11 on df = 84, $\chi^{2}$:df = 3.56) although chi-square ratio was outside the acceptable range. Modification indices suggested that adding a correlated residual between the two core items in the social defeat factor (56.832) would improve model fit (CFI = 0.95, TLI = 0.94, RMSEA = 0.07 (CI 0.06–0.08), $\chi^{2}$ = 244.59 on df = 83, $\chi^{2}$:df = 2.95) but this was not applied as it added complexity to the model which was marginally acceptable without the modification. Superior fit for the four-factor model was confirmed by chi-square comparison with the three-factor model ($\Delta$χ^2^ = 76.06, $\Delta$df = 3, p ≤ .001).

There were positive correlations among all four factors which loaded together onto a second-order factor with acceptable fit on three indices (CFI = 0.94, TLI = 0.92, RMSEA = 0.08 (CI 0.07–0.09) and chi-square ratio slightly above the acceptable range ($\chi^{2}$ = 303.94 on df = 86, $\chi^{2}$:df = 3.53). Model fit was not significantly different from the four-factor model ($\Delta$χ^2^ = 3.146, $\Delta$df = 3, p = .207) suggesting that a combined total score is meaningful alongside the individual factor scores. Factor loadings are shown in Table 2.

**Table 2:** Bullied Cognitions Inventory (BCI) factor loadings and correlations

Item		Factors							
		1		2		3		4	
		EFA	CFA	EFA	CFA	EFA	CFA	EFA	CFA
11	If I told someone I was picked on they would think I am weak	0.937	0.868						
12	It is shameful to have been picked on	0.540	0.687						
13	If people knew I was picked on they would think less of me	0.725	0.900						
23	I need to prove to myself I’m different from what I was then			0.783	0.673				
15	My reaction to being picked on is overdramatic			0.689	0.672				
16	It is embarrassing that I still think about it			0.561	0.785				
19	It is unreasonable to feel bad as much worse things can happen			0.686	0.630				
20	It is my fault that I am not moving on			0.665	0.781				
22	I need to succeed to show I’m better than them			0.631	0.772				
4	People see me as the type of person who gets picked on					0.900	0.815		
14	When I meet someone new they can sense that I’ve been picked on					0.718	0.771		
21	I’m likely to be picked on again					0.679	0.774		
8	I have been totally degraded							0.877	0.714
17	I have been completely humiliated							0.704	0.693
5	If I hadn’t been picked on maybe I would be a better person							0.166	0.746
	Inter factor correlations								
	Factor 1: “Degraded in the eyes of others”	–	–						
	Factor 2: “Negative interpretations of reactions to bullying”	0.731	0.811	–	–				
	Factor 3: “Recognisable as a bullying victim”	0.732	0.766	0.669	0.771	–	–		
	Factor 4: “Social defeat”	0.711	0.796	0.704	0.898	0.662	0.856	–	–
	Second-order factor loadings	–	0.876	–	0.911	–	0.868	–	0.843
	Outcome correlations								
	Social anxiety	0.483	0.452	0.499	0.515	0.492	0.538	0.504	0.479
	PTSD	0.558	0.518	0.607	0.580	0.557	0.505	0.643	0.601
	Depression	0.500	0.438	0.526	0.553	0.471	0.479	0.555	0.537
	Anxiety	0.522	0.471	0.541	0.532	0.531	0.497	0.587	0.541

*EFA* exploratory factor analysis, *CFA* confirmatory factor analysis. Item numbers refer to measure development phase and differ from item numbering in the final measure. Social anxiety (SPIN; range 0–68). PTSD to worst bullying experience (PCL-5; range 0–80). Depression (PHQ-9; range 0–27). General anxiety (GAD-7; range 0–21).

### Reliability and Validity

Reliability assessments within the CFA sample showed that internal consistency was very good for the factors (Cronbach’s $\alpha$ = 0.82–0.85) and full scale ($\alpha$ = 0.93). Test–retest reliability was excellent for the total scale (r = 0.88) and excellent or good for individual factors (1: r = 0.76, 2: r = 0.84, 3: r = 0.80, 4: r = 0.68). Item deletion statistics based on alpha did not support dropping any items. Convergent validity was partially confirmed such that percent variance explained by extracted factors was above 0.5 for items other than item 12 (*r*^*2*^ = 0.472), item 15 (*r*^*2*^ = 0.451), item 19 (*r*^*2*^ = 0.397), item 22 (*r*^*2*^ = 0.419), item 23 (*r*^*2*^ = 0.453). Discriminant validity was marginally acceptable with factor correlations between 0.766 and 0.898. Criterion validity for the full scale was established by significant medium-large Pearson correlations with symptoms of social anxiety (*r* = 0.57) and PTSD (*r* = 0.65). Factor and outcome correlations are in Table 2.

The BCI total score had large correlations with existing cognitive measures, PTCI (*r* = 0.78) and SAQ (*r* = − 0.72; higher scores on the SAQ indicate lower endorsement of unhelpful beliefs). To test the additional contribution of the BCI in explaining variance in PTSD and social anxiety symptoms, multiple linear regression analysis was conducted including BCI total score, presence of power imbalance, and PTCI or SAQ respectively. The models explained 59.8% of the variance in PTSD symptoms (R^2^ = .598, R^2^_Adjusted_ = .596, *F*(3, 768) = 380.220, *p* < .001 and 41.7% of the variance in social anxiety symptoms (R^2^ = .417, R^2^_Adjusted_ = .403, *F*(3, 125) = 29.835, *p* < .001). The BCI explained small but significant additional variance in PTSD symptoms over the PTCI (11.6%) but the contribution to explaining social anxiety symptoms over the SAQ (13.7%) was not significant as the sample size was smaller. To check for potential multicollinearity in the model, variance inflation factors were evaluated and were not excessive. See Table 3.

**Table 3:** Multiple regressions of Bullied Cognitions Inventory (total score) with PTCI on PTSD symptoms and SAQ on social anxiety symptoms

	Social anxiety (*n* = 129)				PTSD (*n* = 772)			
	B	SE	β	p	B	SE	β	p
Constant	46.822	12.375		< .001	− 11.608	3.521		.001
Power imbalance	3.220	4.892	.046	.512	2.440	1.789	.031	.173
SAQ	− 6.748	1.355	− .493	< .001	–	–	–	–
PTCI	–	–	–	–	.533	.029	.663	< .001
BCI	.137	.075	.182	.071	.116	.033	.130	< .001

Social anxiety symptoms measured using SPIN. PTSD symptoms to worst bullying experience measured using PCL-5

*Power imbalance determined using CBVS-R.* *PTCI* Posttraumatic Cognitions Inventory; completed by 772 participants, *SAQ* Social Attitudes Questionnaire; completed by 129 participants, *BCI* Bullied Cognitions Inventory

### Group Comparisons

Participants were classified by probable SAD or PTSD diagnoses. In total, 173 of the 854 participants (20.3%) were below the symptom thresholds for probable diagnosis of both SAD and PTSD, 315 (36.9%) met the threshold for SAD only, 43 (5.0%) for PTSD only, and 323 (37.8%) for both SAD and PTSD combined.

The BCI predicted diagnostic group membership (χ^2^ (3, N = 835) = 377.58, Nagelkerke R^2^ = .40, *p* < .001) and distinguished between the non-clinical and other groups, such that higher scores were associated with membership of one of the clinical groups. Compared with the non-clinical group, higher BCI significantly increased the likelihood being in the SAD alone group (OR 1.06, 95% CI [1.04, 1.07]), PTSD alone group (OR 1.07, 95% CI [1.05, 1.09]), and the combined SAD + PTSD group (OR 1.12, 95% CI [1.10, 1.14]). Within the clinical groups, there was no distinction between the SAD alone and PTSD alone groups (OR 1.01, 95% CI [0.99, 1.03]) but an increase in BCI score significantly increased the likelihood of being in the SAD + PTSD group compared with SAD alone (OR 1.06, 95% CI [1.05, 1.07]) or PTSD alone (OR 1.05, 95% CI [1.03, 1.07]).

## Discussion

The Bullied Cognitions Inventory (BCI) is a reliable and valid measure of beliefs about self and others related to bullying experiences that cluster into four factors, “degraded in the eyes of others” (3 items), “negative interpretations of reactions to bullying” (6 items), “recognisable as a bullying victim” (3 items), and “social defeat” (3 items). Scores on this measure explained significant variance in social anxiety and PTSD symptoms experienced by young people with a history of bullying. Among those suffering with social anxiety, the measure distinguishes those also experiencing high symptoms of PTSD related to their bullying experiences and vice versa. The BCI could be a useful tool for assessment and monitoring in cognitive therapy for people who experience ongoing distress related to experiences of bullying. It added additional variance in explaining PTSD symptoms over and above the PTCI, with a similar effect size for explaining additional variance in social anxiety over and above the SAQ not quite reaching significance because of the smaller sample size.

Correlations among the clusters of beliefs about self and others related to bullying experiences in the BCI reflect strong potential links between the concepts that may reinforce each other. For example, beliefs about having been degraded by bullying experiences are entwined with beliefs that if others found out about what happened then social wellbeing could be damaged. In addition, beliefs that past events have the power to mark you out and make you permanently recognisable as a target for bullying are held alongside self-critical thoughts that those experiences were not serious enough to warrant a reaction. Taken together, these beliefs may increase perceived social threat. Clinically, the BCI total score provides an indication of severity of unhelpful cognitions that are associated with social anxiety and PTSD symptoms. In addition, subscale and individual item scores can be used to identify particular targets for intervention. Of note, in addition to social anxiety and PTSD, scores on the BCI were also significantly correlated with depression and general anxiety so it is possible that the measures may represent cognitions and behaviours related to bullying that drive symptoms of a range of disorders.

Features of the study may limit specificity of conclusions. First, the sample was not selected according to symptom levels and included participants with low and high scores across all symptom domains, potentially inflating correlations and obscuring clinically interesting symptom patterns among those with higher scores. Of note, relatively fewer participants were categorised as experiencing PTSD-only, with far more experiencing social anxiety alone or in combination with PTSD. This reflects the likely clinical picture among young people who were bullied but means that PTSD-only group comparisons were underpowered. Future research could use network analyses to explore whether certain subgroups of symptoms (rather than total scores for social anxiety, PTSD, anxiety, and depression) show stronger associations to specific cognitive themes. Second, items within the measure could be further refined, particularly within the “*social defeat*” factor where the item, *“If I hadn’t been picked on maybe I would be a better person”* was retained due its relative position in the model structure and contribution to model stability. Future research could adapt the wording to be more direct, for example *“being picked on made me an inferior person”*, and reassess variance explained within the factor and contribution to overall measure stability. Finally, data collection method and sample characteristics could be important. Data were self-reported online potentially compromising validity despite data checks. Although the sample was diverse, notably in terms of sexual orientation and ethnicity, people who identified as female were overrepresented and all were prospective students. Furthermore, the sample was self selected and it is possible that those with experiences of bullying and negative effects were more interested in taking part. Therefore, findings may not generalise to younger or older people, or people with very different circumstances, and replication is needed in a gender-balanced non-selected sample.

There are clinical implications for the assessment of bullying experiences and related cognitions. Social anxiety symptoms were high in this sample of young adults with a history of bullying and, of those with clinically relevant social anxiety symptoms, just over half (51.2%) also had clinically relevant PTSD symptoms to their worst bullying experience. The extent of clinically relevant PTSD symptoms was therefore even higher in this sample than in another sample of adults with experiences of social trauma (Bjornsson et al., 2020) in which just under a third (32.7%) of individuals with SAD also reported clinically significant symptoms of posttraumatic stress. The high level of symptoms is perhaps unsurprising given the nature of the sample as all having experienced bullying and theoretical likelihood that both types of symptoms were precipitated or exacerbated by shared experiences of socially traumatic events. Only one in five participants did not reach the threshold for likely clinical diagnosis of SAD or PTSD. The PTSD alone group was by far the smallest group, with nearly seven times fewer participants than in each of the other clinical groups, SAD alone and SAD + PTSD. The temporal associations between SAD and PTSD are unclear (Collimore et al., 2010) but high comorbidity suggests that mechanisms and pathways to symptoms may overlap among people who have experienced the social trauma of bullying. It is possible that people who have been bullied and are experiencing SAD and PTSD-like symptoms related to their experiences may be best understood as experiencing a stress response to traumatic social experiences that involves an ongoing sense of social threat.

The high rate of symptoms of both social anxiety and PTSD in this sample supports ongoing focus on bullying experiences as psychologically impactful events. Clinicians who work with bullied people should be aware of the potential for PTSD-like symptoms related to their experiences. If used within social anxiety treatment, a high score could suggest using techniques associated with processing negative past experiences, such as imagery rescripting and updating memories related to the bullying experiences to challenge unhelpful beliefs connected to (the worst parts of) the experience (Wild & Clark, 2011). The utility of routine assessment of bullying is supported by themes identified in this measure that may lead young people to avoid disclosure, such as shame (e.g., “*If I told someone I was picked on they would think I am weak”*) and self-criticism particularly in terms of downplaying the seriousness of the events (e.g., “*It is unreasonable to feel bad as much worse things can happen*”). The BCI is a bespoke measure of beliefs related to bullying that may be of particular relevance for young people who are experiencing symptoms of social anxiety and PTSD related to their experiences.

### Supplementary Information

Below is the link to the electronic supplementary material.

Supplementary file1 (DOCX 33 KB)

## Data Availability

The datasets generated during and/or analysed during the current study are available from the corresponding author on reasonable request.
