# Supplementary material for: Development and Validation of the Bullied Cognitions Inventory (BCI)
Source: Cognit Ther Res. 2023 Sep 7;47(6):1033–45. doi: 10.1007/s10608-023-10412-6 (PMC10620262; doi:10.1007/s10608-023-10412-6)
Supplement: Supplementary file 1 — Supplementary file1 (DOCX 33 KB) [file 10608_2023_10412_MOESM1_ESM.docx]

Supplementary materials.
Pearson correlations of all items in Bullied Cognitions Inventory (BCI) item pool and final BCI total score.

| Item | **1** | 2 | **3** | 4 | 5 | 6 | **7** | 8 | 9 | 10 | 11 | 12 | 13 | 14 | 15 | 16 | 17 | 18 | 19 | 20 | 21 | 22 | 23 |
| --- | --- | --- | --- | --- | --- | --- | --- | --- | --- | --- | --- | --- | --- | --- | --- | --- | --- | --- | --- | --- | --- | --- | --- |
| **1.** | **--** |  |  |  |  |  |  |  |  |  |  |  |  |  |  |  |  |  |  |  |  |  |  |
| **2.** | **.557** | -- |  |  |  |  |  |  |  |  |  |  |  |  |  |  |  |  |  |  |  |  |  |
| **3.** | **.402** | **.488** | **--** |  |  |  |  |  |  |  |  |  |  |  |  |  |  |  |  |  |  |  |  |
| 4. | **.476** | **.567** | **.751** | -- |  |  |  |  |  |  |  |  |  |  |  |  |  |  |  |  |  |  |  |
| 5. | **.415** | **.547** | **.492** | .501 | -- |  |  |  |  |  |  |  |  |  |  |  |  |  |  |  |  |  |  |
| **6.** | **.488** | **.622** | **.464** | **.525** | **.571** | -- |  |  |  |  |  |  |  |  |  |  |  |  |  |  |  |  |  |
| **7.** | **.607** | **.478** | **.402** | **.447** | **.318** | **.369** | **--** |  |  |  |  |  |  |  |  |  |  |  |  |  |  |  |  |
| 8. | **.465** | **.544** | **.477** | .499 | .553 | **.557** | **.390** | -- |  |  |  |  |  |  |  |  |  |  |  |  |  |  |  |
| **9.** | **.545** | **.630** | **.538** | **.629** | **.560** | **.504** | **.519** | **.560** | -- |  |  |  |  |  |  |  |  |  |  |  |  |  |  |
| **10.** | **.495** | **.571** | **.491** | **.582** | **.519** | **.525** | **.476** | **.543** | **.750** | **--** |  |  |  |  |  |  |  |  |  |  |  |  |  |
| 11. | **.484** | **.584** | **.494** | .570 | .540 | **.516** | **.406** | .537 | **.727** | **.760** | -- |  |  |  |  |  |  |  |  |  |  |  |  |
| 12. | **.499** | **.543** | **.455** | .506 | .505 | **.500** | **.411** | .563 | **.648** | **.600** | .638 | -- |  |  |  |  |  |  |  |  |  |  |  |
| 13. | **.504** | **.563** | **.484** | .545 | .545 | **.522** | **.483** | .557 | **.786** | **.783** | .782 | .640 | -- |  |  |  |  |  |  |  |  |  |  |
| 14. | **.493** | **.572** | **.625** | .696 | .510 | **.508** | **.493** | .501 | **.624** | **.596** | .549 | .490 | .564 | -- |  |  |  |  |  |  |  |  |  |
| 15. | **.446** | **.506** | **.440** | .430 | .439 | **.487** | **.387** | .415 | **.494** | **.468** | .476 | .426 | .499 | .419 | -- |  |  |  |  |  |  |  |  |
| 16. | **.452** | **.568** | **.447** | .485 | .562 | **.573** | **.312** | .532 | **.547** | **.572** | .585 | .531 | .571 | .466 | .559 | -- |  |  |  |  |  |  |  |
| 17. | **.450** | **.531** | **.496** | .495 | .537 | **.546** | **.363** | .754 | **.558** | **.561** | .561 | .587 | .536 | .506 | .424 | .558 | -- |  |  |  |  |  |  |
| **18.** | **.378** | **.494** | **.464** | **.456** | **.473** | **.468** | **.370** | **.499** | **.512** | **.461** | **.457** | **.431** | **.468** | **.473** | **.394** | **.473** | **.448** | **--** |  |  |  |  |  |
| 19. | **.385** | **.408** | **.345** | .382 | .390 | **.459** | **.304** | .352 | **.403** | **.449** | .436 | .403 | .453 | .351 | .486 | .526 | .390 | **.320** | -- |  |  |  |  |
| 20. | **.500** | **.561** | **.438** | .490 | .525 | **.586** | **.356** | .538 | **.497** | **.497** | .532 | .493 | .525 | .476 | .589 | .634 | .524 | **.439** | .531 | -- |  |  |  |
| 21. | **.446** | **.534** | **.619** | .655 | .536 | **.541** | **.400** | .531 | **.553** | **.543** | .528 | .458 | .538 | .580 | .438 | .490 | .537 | **.439** | .396 | .515 | -- |  |  |
| 22. | **.295** | **.467** | **.359** | .389 | .457 | **.518** | **.229** | .420 | **.419** | **.448** | .451 | .422 | .452 | .345 | .400 | .443 | .401 | **.391** | .376 | .424 | .402 | -- |  |
| 23. | **.351** | **.550** | **.405** | .426 | .502 | **.566** | **.242** | .475 | **.485** | **.501** | .494 | .483 | .478 | .400 | .442 | .562 | .483 | **.410** | .451 | .537 | .409 | .588 | -- |
| Total | .**605** | **.732** | .**667** | .725 | .740 | **.728** | **.495** | .749 | **.770** | **.763** | .789 | .746 | .792 | .704 | .679 | .781 | .756 | **.599** | .633 | .761 | .724 | .638 | .714 |

*Note.* All correlations are significant, *p* < .001. Item numbering refers to scale development phase and differs from numbering in the final measure. Items 1, 2, 3, 6, 7, 9, 10, 18 were not included in the final measure.

Graham, B. & Ehlers, A. (2023). Development and Validation of the Bullied Cognitions Inventory (BCI). *Cognitive Therapy and Research.*
